# Distinct Populations of Hepatic Stellate Cells in the Mouse Liver Have Different Capacities for Retinoid and Lipid Storage

**DOI:** 10.1371/journal.pone.0024993

**Published:** 2011-09-16

**Authors:** Diana N. D'Ambrosio, José L. Walewski, Robin D. Clugston, Paul D. Berk, Richard A. Rippe, William S. Blaner

**Affiliations:** 1 Department of Medicine, College of Physicians and Surgeons, Columbia University, New York, New York, United States of America; 2 Institute of Human Nutrition, College of Physicians and Surgeons, Columbia University, New York, New York, United States of America; 3 Division of Gastroenterology and Hepatology, Department of Medicine, University of North Carolina at Chapel Hill, Chapel Hill, North Carolina, United States of America; University of Montreal, Canada

## Abstract

Hepatic stellate cell (HSC) lipid droplets are specialized organelles for the storage of retinoid, accounting for 50–60% of all retinoid present in the body. When HSCs activate, retinyl ester levels progressively decrease and the lipid droplets are lost. The objective of this study was to determine if the HSC population in a healthy, uninjured liver demonstrates heterogeneity in its capacity for retinoid and lipid storage in lipid droplets. To this end, we utilized two methods of HSC isolation, which leverage distinct properties of these cells, including their vitamin A content and collagen expression. HSCs were isolated either from wild type (WT) mice in the C57BL/6 genetic background by flotation in a Nycodenz density gradient, followed by fluorescence activated cell sorting (FACS) based on vitamin A autofluorescence, or from collagen-green fluorescent protein (GFP) mice by FACS based on GFP expression from a GFP transgene driven by the collagen I promoter. We show that GFP-HSCs have: (i) increased expression of typical markers of HSC activation; (ii) decreased retinyl ester levels, accompanied by reduced expression of the enzyme needed for hepatic retinyl ester synthesis (LRAT); (iii) decreased triglyceride levels; (iv) increased expression of genes associated with lipid catabolism; and (v) an increase in expression of the retinoid-catabolizing cytochrome, CYP2S1. Conclusion: Our observations suggest that the HSC population in a healthy, uninjured liver is heterogeneous. One subset of the total HSC population, which expresses early markers of HSC activation, may be “primed” and ready for rapid response to acute liver injury.

## Introduction

Retinoids (vitamin A and its metabolites, both natural and synthetic) are essential to many physiological processes, including reproduction, embryonic development, bone growth, immunity and vision [1]–[3]. Seventy percent of retinoid in the body is stored in the liver [4], [5], and, of this fraction, 90–95% is stored in lipid droplets within hepatic stellate cells (HSCs) [6]. These lipid droplets are a distinguishing feature of HSCs and have been proposed to be specialized organelles for retinoid storage due to their retinoid content and responsiveness to dietary retinoid status [7]. Retinyl esters comprise approximately 40% of the lipids present in these droplets, more than any other single lipid species [8], [9]. And while the remaining 60% is all non-retinoid lipid species, including triglyceride, cholesterol ester, cholesterol, phospholipid and free fatty acid, there is considerable data in the literature suggesting that the formation and maintenance of these lipid droplets are retinoid-dependent processes [7].

Moriwaki *et al.* showed that the lipid composition of HSC lipid droplets is strongly regulated by dietary retinoid status, but not by dietary triglyceride intake [8]. HSC lipid droplet retinoid lipid species are decreased in response to a low retinol diet and both retinoid and non-retinoid lipids are elevated in response to a high retinol diet; however, neither retinoid nor non-retinoid content is affected by low or high fat diets. Other data that are highly suggestive of the role of retinoids in these droplets relates to the enzyme lecithin∶retinol acyltransferase (LRAT), which is the only known enzyme in the liver capable of synthesizing retinyl ester. O'Byrne *et al.* showed that the HSCs of wild type (WT) mice have large, distinct lipid droplets, but the cells of LRAT-null mice have none [10]. Thus, the ability to synthesize and store retinyl ester in HSCs is necessary for the presence of HSC lipid droplets.

It is well-established that when HSCs activate, in response to a hepatic insult or disease, the HSCs loose their lipid droplet content and undergo a simultaneous decrease in retinyl ester levels. Leo and Lieber found that there is a nearly 5-fold decrease in total hepatic retinol levels with the development of alcoholic hepatitis and another approximately 4-fold decrease with the development of cirrhosis [11]. It has also been shown in cultured HSCs that retinyl ester stored in HSC lipid droplets is first hydrolyzed and then released into the media as retinol [12]. As HSCs transition from a quiescent to a myofibroblastic phenotype, they undergo increased extracellular matrix production, including increased synthesis of collagen I, and become fibrogenic [13], [14]. It has not yet been unequivocally determined whether the loss of HSC lipid droplets is a cause or consequence of activation.

We are interested in understanding the factors that regulate HSC retinoid storage as retinyl esters in lipid droplets and the factors that regulate HSC lipid droplet genesis and dissolution. In this study, we employ two methods of HSC isolation, which leverage distinct properties of these cells. One method relies on HSC lipid droplet vitamin A content and the other on HSC expression of collagen I. It was recently shown that the changes in gene expression that accompany HSC activation and the loss of retinyl ester lipid droplets are regulated differently in the *in vitro* and *in vivo* models of activation [15]. Similarly, a goal of this study is to determine whether different methods of HSC isolation will yield phenotypically distinct populations of cells and how this may reflect heterogeneity of HSCs in the liver at a given time. We are particularly interested in how these populations compare with regard to their capacities for retinoid storage and lipid droplet formation. Our findings are presented below.

## Materials and Methods

### Animals

WT and collagen-green fluorescent protein (GFP) mice were used, with both strains congenic for the C57BL/6 genetic background. The collagen-GFP mice have been previously described [16]. Briefly, a gene construct was made containing 3,122 bp of the α1 (I) collagen (*Col1a1*) gene promoter linked to the GFP reporter gene [16]. Hepatic expression of collagen I is solely in HSCs [17], [18], and its expression is increased as the cells become activated [19], [20]. Thus, GFP expression from this transgene can be used to identify and isolate HSCs. All mice used in the study were males between 90 and 120 days of age at the time of sacrifice. Animals were allowed *ad libitum* access to water and a standard nutritionally complete rodent chow diet (W. F. Fisher and Sons, Inc., Somerville, NJ). All mice were maintained on a 12-h dark-light cycle, with the period of darkness between 7:00 a.m. and 7:00 p.m. in a conventional barrier facility. The animal experiments described in this report were conducted in accordance with the National Research Council's Guide for the Care and Use of Laboratory Animals and were approved by the Columbia University Institutional Committee on Animal Care (IACUC Protocol # AC-AAAA9687).

### Hepatic fibrosis induction

WT C57BL/6 mice were given weekly injections of either 0.5 µl carbon tetrachloride (CCl_4_) per gram body weight administered in corn oil or an equivalent volume of corn oil alone for 4 weeks and then sacrificed. Liver tissue was collected and stored immediately at −80°C prior to analysis.

### Hepatocyte isolations

Livers were perfused *in situ* with EGTA for 5 min and collagenase D (0.5 mg/ml, Roche Diagnostics, Indianapolis, IN) for 15 min, respectively, at a flow rate of 5 ml/min. After perfusion, the partially digested liver was excised, the digest passed through a 100 µm nylon mesh to remove undigested materials, and resuspended in Dulbecco's Modified Eagle Medium (DMEM; Gibco, Grand Island, NY) containing 1% penicillin/streptomycin. Hepatocytes were separated from the non-parenchymal cells and debris by centrifugation at 4°C in the following sequence: twice for 5 min at 20 *g*, once for 10 min at 50 g, and twice again for five min at 20 g. The supernatant was aspirated and the hepatocytes present in the pellet were resuspended in DMEM. Cell yields were determined by counting on a hemacytometer.

### HSC isolations

Primary mouse HSCs were isolated according to established protocols [9], [21], [22]. Briefly, livers were perfused *in situ* with EGTA for 5 min, pronase E (0.4 mg/ml, EMD Chemicals Inc., Gibbstown, NJ) for 5 min and collagenase D (0.5 mg/ml, Roche Diagnostics) for 8 min, respectively, at a flow rate of 5 ml/min. After excision of the liver from the body, the liver digests were filtered through a cell strainer and washed with Gey's Balanced Salt Solution (Sigma, St. Louis, MO) containing DNase I (2 mg/ml, Roche Diagnostics). For the WT C57 mice, HSCs were purified from the remainder of non-parenchymal cells and hepatocyte-derived debris by floatation through 9% (w/v) Nycodenz (Axis-Shield PoC AS, Oslo, Norway) in Gey's Balanced Salt Solution (without NaCl). Subsequently, the cells were separated by FACS using FACS Calibur (Becton Dickinson, Franklin Lakes, NJ), and vitamin A auto-fluorescent cells were collected based on their emission at 460 nm. This method of HSC isolation yields approximately 10% of the total HSCs estimated to be in the liver. For the collagen-GFP mice, parenchymal cells and debris were separated from non-parenchymal cells by centrifugation at 50 g for 2 minutes. The remaining non-parenchymal cell suspension was then separated by FACS, and GFP-positive cells were collected based on their emission at 530 nm. This method of HSC isolation yields approximately 10% of the total HSCs estimated to be in the liver.

### Isolation of RNA and cDNA synthesis

Total RNA was isolated from HSCs using the Qiagen RNeasy kit (Qiagen, Valencia, CA). cDNA synthesis and amplification were performed using the NuGEN WT-Ovation Pico RNA Amplification kit (NuGEN, San Carlos, CA). The resulting cDNA was then purified using the Qiagen QIAquick PCR Purification kit (Qiagen). cDNA fragmentation and labeling were performed using the NuGEN FL-Ovation cDNA Biotin Module V2 kit (NuGEN).

### DNA microarray analysis

Array analysis was conducted on the Affymetrix Mouse Genome 430 2.0 Array (Affymetrix, Santa Clara, CA) and analyzed using the GeneSifter gene expression analysis suite (Geospiza, Seattle, WA). Individual gene expression values were normalized to the median. Significant changes in gene expression between sample sets were identified by a Student's *t*-test and subsequent Benjamini–Hochberg correction, with a p-value of 0.05 as the criterion for statistical significance. All microarray data are MIAME compliant and are deposited as ArrayExpress accession E-MEXP-3231.

### Quantitative real-time PCR

Differential gene expression was confirmed by quantitative real-time polymerase chain reaction (qRT-PCR) using commercially available primer-probe sets (**Table S1**, Applied Biosystems, Foster City, CA). qRT-PCR was performed on a LightCycler 480 (Roche Diagnostics).

### Immunohistochemistry

Liver tissue samples from collagen-GFP mice were collected into 10% formalin. Immunohistochemistry was conducted by the Herbert Irving Comprehensive Cancer Center Histology Service using standard protocols. Desmin expression was localized using an anti-desmin monoclonal mouse anti-human antibody at a dilution of 1∶400 (Dako, Carpinteria, CA, clone D33, IR606), and GFP was localized using an anti-GFP rabbit polyclonal antibody at a dilution of 1∶500 (Invitrogen, Carlsbad, CA, A-11122). Fluorescent images were captured at 40× magnification using a Zeiss Axiovert 200 M Microscope with Apotome (Zeiss, Göttingen, DE), and images were taken using an AxioCam MRm camera (Zeiss).

### Primary HSC culture

HSCs were isolated from WT and collagen-GFP mice as described above. Approximately 0.5×10^6^ cells were resuspended in DMEM containing 10% fetal bovine serum and 1% penicillin/streptomycin, seeded on 35 mm glass bottom dishes (MatTek Co., Ashland, MA) and incubated at 37°C overnight. Phase contrast and fluorescent images were captured using an FSX100 Olympus Microscope (Olympus America Inc., Center Valley, PA).

### Reverse-phase HPLC analysis

Liver and HSC retinyl ester levels were determined by procedures described previously [10]. Briefly, 200 mg liver tissue was homogenized in 2 ml of PBS (10 mM sodium phosphate, pH 7.2, 150 mM sodium chloride) using a Polytron homogenizer (Brinkmann Instruments, Westbury, NY) and 200 µl aliquots were taken for further analysis. For HSCs, aliquots of 100,000 cells were resuspended in 1 ml PBS. All samples were then treated with an equal volume of absolute ethanol containing a known amount of retinyl acetate as an internal standard, and the retinoids present in the homogenates were extracted into hexane. The extracted retinoids were separated on a 4.6×250 mm Ultrasphere C_18_ column (Beckman, Fullerton, CA) preceded by a C_18_ guard column (Supelco, Bellefonte, PA), using 70% acetonitrile, 15% methanol, 15% methylene chloride as the running solvent flowing at 1.8 ml/min. Retinol and retinyl esters (retinyl palmitate, oleate, linoleate, and stearate) were detected at 325 nm and identified by comparing the retention times and spectral data of experimental compounds with those of authentic standards. Concentrations of retinol and retinyl esters in the tissues were quantitated by comparing integrated peak areas for each retinoid against those of known amounts of purified standards. Loss during extraction was accounted for by adjusting for the recovery of the internal standard added immediately after homogenization of the cells and tissues.

### Triglyceride analysis

Approximately 200 mg of liver or 0.5×10^6^ HSCs were homogenized in 1 ml of PBS using a Polytron homogenizer. The lipids in the homogenates were then extracted into chloroform∶methanol (2∶1 v/v), and the lower, chloroform phase was collected. The upper phase was re-extracted with an additional volume of chloroform∶methanol to ensure complete triglyceride recovery, and the pooled chloroform phases were evaporated under N_2_. One milliliter of 2% Triton X-100 in chloroform for liver or 50 µl for HSCs was then added to the samples, and the chloroform was re-evaporated under N_2_. For liver, triglycerides were solubilized for colorimetric assay through addition of 1 mL of deionized water into the glass tubes. Employing a Matrix Plus Chemistry Reference Standard (Verichem Laboratories Inc., Providence, RI), according to the manufacturer's instructions, a colorimetric triglyceride assay using the Infinity Triglyceride Reagent (Thermo Fisher Scientific Inc., Middletown, VA) was performed in a 96-well plate for 5 µl of each liver sample. For HSCs, 200 µl of Triglyceride Reagent was added directly to the dried-down samples, and the entire volume was read in the colorimetric assay. Color development was measured on a Multiskan Plus Microtiter Plate Reader at 520 nm.

### Western blot protein analysis

HSC proteins were analyzed by Western blot assay. For all proteins analyzed, 5 µg of total protein was separated on 10% SDS–PAGE gels and transferred onto polyvinylidene fluoride membrane (Millipore Immunobilon-P Transfer Membrane) at 100 V for 1 h at 4°C. The membranes were then incubated with 10% milk blocking buffer for 1 h at room temperature (RT), followed by overnight incubation at 4°C with the following primary antibodies: alpha smooth muscle actin (1∶100, rabbit polyclonal, Abcam, Cambridge, MA, ab5694), adipose differentiation related protein (1∶500, rabbit polyclonal, Abcam, ab52356), CYP2S1 (1∶500, rabbit polyclonal, Abcam, ab69650), CYP2E1 (1∶500, rabbit polyclonal, Abcam, ab28146) and LRAT (1∶500, mouse monoclonal, gift from Dr. Krzysztof Palczewski, Department of Pharmacology, Case Western Reserve University). The secondary antibody incubations were for 1 h at RT with either a donkey horseradish peroxidase-conjugated anti-rabbit IgG antiserum (1∶10,000, GE Healthcare, Piscataway, NJ, cat# NA934V) or goat horseradish peroxidase-conjugated anti-mouse IgG antiserum (1∶10,000, EMD Chemicals Inc., Gibbstown, NJ, cat# 401215). Immunoblots were developed using the ECL system (Thermo Scientific, Rockford, IL).

### Statistical analysis

All data are presented as means ± S.D. A Student's *t*-test was used to analyze for statistically significant differences between groups. Groups were considered to be significantly different when p<0.05.

## Results

### Methods of HSC isolation

**Figure 1:**
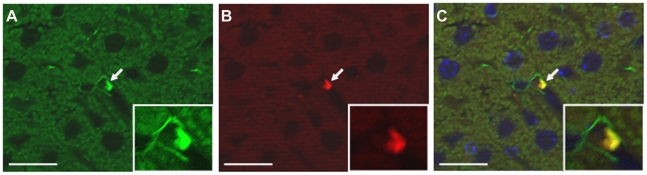
Co-localization of GFP expression with expression of the HSC marker desmin. Immunohistochemistry was performed on sections of liver from collagen-GFP mice using antibodies to the HSC marker desmin (A) and GFP (B). Merged image shows colocalization of desmin and GFP expression (C). DAPI (blue) staining of nuclei is also shown in the merged image. Micrographs are at 40× magnification. Scale bars, 15 µm. Arrows point to a hepatic stellate cell. Insets show magnified view of one HSC.

To obtain a relatively vitamin A-enriched population of HSCs, we conducted FACS on HSCs obtained by conventional Nycodenz flotation of cells present in a pronase digest of a liver. Vitamin A autofluoresces at 460 nm upon excitation at 350 nm, so exposure to this excitation wavelength allowed us to collect a population of cells that is enriched in vitamin A. This population of lipid droplet- and vitamin A-enriched HSCs will be referred to subsequently as AF-HSCs. The second method of HSC isolation used in this study capitalizes on transgene GFP expression driven by the *Col1a1* promoter in these mice [16] and data indicating that expression of collagen I in the liver occurs exclusively in the HSCs [17], [18]. We performed FACS on digests of non-parenchymal cells and collected GFP-positive cells based on their excitation at 488 nm. This GFP-expressing population of HSCs will be referred to as GFP-HSCs. To confirm that GFP expression is confined to HSCs, we performed immunohistochemistry using antibodies to GFP and the HSC marker desmin [23]. We show that GFP expression is found only in the HSCs, with GFP expression co-localizing with desmin expression, and is not found in the other major cell types in the liver (**Figure 1A–C**). We further analyzed the integrity of these populations by qRT-PCR using desmin mRNA expression as a marker of HSC purity, and we show that the two different methods of HSC isolation yield cell populations with equivalent desmin expression (**Figure S1A**).

### Microarray analysis overview

For this study, we performed gene microarray analysis on populations of AF-HSCs isolated from 6 mice and GFP-HSCs isolated from 5 mice. cDNA purified from these populations was run on Affymetrix Mouse Genome 430 2.0 Arrays, covering over 45,000 transcripts in the mouse genome, and data was analyzed using GeneSifter software. The quality of the samples used is demonstrated by the box plot and scatter plot summaries of the AF and GFP groups, which show that the spread of all the array data points is identical in both groups (**Figure S1B and S1C**). Five thousand three hundred sixty three probe sets were more than 2-fold up- or down-regulated in the GFP-HSCs compared to AF-HSCs (**Table 1**). As a second-stage filter and an analysis with biological implications, the list of transcripts with significant differential expression between AF- and GFP-HSCs was screened against >200 known KEGG (Kyoto Encyclopedia of Genes and Genomics) pathways. This analysis identified 15 KEGG pathways where a significant number of genes were either up- or down-regulated (**Table 2**). Significance at the pathway level was defined as a z-score with an absolute value greater than 2. As described by Walewski *et al.*, a positive z-score greater than or equal to 2 indicates that a significant number of genes in the list of differentially expressed genes are significantly up- or down-regulated in the experimental group in that particular pathway [24]. Notably, a number of the enriched KEGG pathways identified by this study have a direct or indirect relationship with the extracellular matrix (ECM), which is a target of deregulation in HSCs that are in the early stages of activation [13]. These pathways are classified as cellular adhesion molecules (CAMs), ECM-receptor interaction, focal adhesions, and gap junctions (**Table 2**).

**Table 1:** Summary of differentially expressed genes.

Method	p-value Cutoff	Total Changed	Increased	Decreased
Student's *t*-test	0.05	5363*	2676	2687
	0.01	3563	1901	1662
	0.001	1673	825	848
Welch's *t*-test	0.05	5074	2361	2713
	0.01	2674	1064	1610
	0.001	985	230	755

Affymetrix Mouse 430 2.0 microarray chips were used to search for gene expression differences between AF- and GFP-HSCs. Lists of differentially expressed genes were generated using Student's *t*-tests or Welch's *t*-tests, each with three p-value cut-offs, including 0.05, 0.01 and 0.001. For each comparison, the number of total, increased and decreased differentially expressed genes is shown.

*All further analysis conducted on this set of differentially expressed genes.

**Table 2:** KEGG Pathway Enrichment.

z-score	KEGG Pathway	Gene Set	Total Changed	Up	Down	z-score
UP	Cell adhesion molecules (CAMs)	131	51	34	17	5.64
	Cytokine-cytokine receptor interaction	232	74	48	26	4.95
	Leishmaniasis	66	30	19	11	4.72
	Type I diabetes mellitus	44	19	14	5	4.50
	Leukocyte transendothelial migration	114	44	26	18	4.16
	ECM-receptor interaction	76	27	18	9	3.63
	Maturity onset diabetes of the young	25	10	8	2	3.41
	Steroid biosynthesis	17	6	6	0	3.25
	Glycine, serine and threonine metabolism	30	9	8	1	2.80
DOWN	Axon guidance	127	54	24	30	4.76
	Focal adhesion	189	70	33	37	4.01
	Gap junction	83	25	7	18	3.25
	Cell cycle	123	27	4	23	2.89
	ErbB signaling pathway	84	22	6	16	2.48
	Chemokine signaling pathway	173	47	20	27	2.11

Tests of KEGG pathway enrichment were conducted on the set of 5363 differentially expressed genes identified by the Student's *t*-test as having a p-value equal to or less than 0.05. The tests were performed using the GeneSifter software. A pathway is defined as enriched if it has a positive z-score up or down greater than or equal to 2.

### GFP-HSCs are phenotypically similar to HSCs undergoing early activation

**Figure 2:**
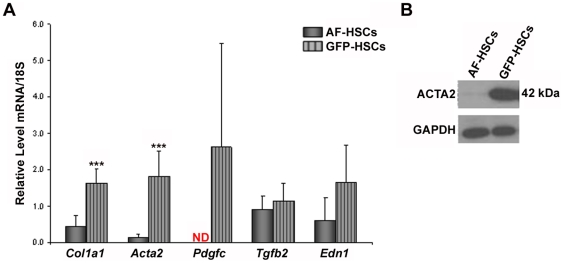
mRNA and protein expression of markers of HSC activation. (A) Relative mRNA expression levels of *Col1a1*, *Acta2*, *Pdgfc*, *Tgfb*, and *Edn1* in AF-HSCs (solid bars) and GFP-HSCs (stripped bars) are shown. Values are normalized to 18S and given as the mean ±1 S.D. for 5 AF and 5 GFP HSC isolations. Significance was determined by a Student's *t*-test, *** p-value<0.001. ND means “not detected.” (B) Protein levels of ACTA2 in AF-HSCs and GFP-HSCs. Experiment was repeated with similar results.

As a result of activation, HSCs undergo increased collagen I production and a simultaneous loss of their retinyl ester-containing lipid droplets [18], [19]. Because we have selected for a population of HSCs that express *Col1a1*, we were interested in whether GFP-HSCs would display characteristics of activated HSCs. The array suggests that a number of markers of HSC activation are elevated in the GFP-HSCs, including alpha smooth muscle actin (*Acta2*), platelet-derived growth factor C (*Pdgfc*), transforming growth factor beta 2 (*Tgfb2*), and endothelin (*Edn1*) (**Table 3**). Elevated expression of all markers was confirmed by qRT-PCR; however, only *Acta2* up-regulation was found to be significant (**Figure 2A**). Elevated protein levels of ACTA2 are also shown by western blot analysis (**Figure 2B**).

**Table 3:** Markers of HSC Activation.

AFFY ID	Gene Name	Gene ID	FC	p-value
1449254_at	Secreted phosphoprotein 1	*Spp1*	**115.5**	0.000001
1419123_a_at	Platelet-derived growth factor, C polypeptide	*Pdgfc*	**39.3**	0.002963
1449351_s_at	Platelet-derived growth factor, C polypeptide	*Pdgfc*	**32.5**	0.003461
1456658_at	Actin, alpha 2, smooth muscle	*Acta2*	**5.5**	0.001718
1456156_at	Leptin receptor	*Lepr*	**4.2**	0.007139
1451924_a_at	Endothelin 1	*Edn1*	**4.2**	0.000544
1416454_s_at	Actin, alpha 2, smooth muscle	*Acta2*	**3.5**	0.016302
1438303_at	Transforming growth factor, beta 2	*Tgfb2*	**2.7**	0.002149
1448729_a_at	Septin 4	*Sept4*	**0.49**	0.003582
1443434_s_at	Plexin C1	*Plxnc1*	**0.45**	0.000309
1455851_at	Bone morphogenetic protein 5	*Bmp5*	**0.40**	0.000004

Differences in gene expression are reported as fold-change (FC) relative to AF-HSCs.

**Figure 3:**
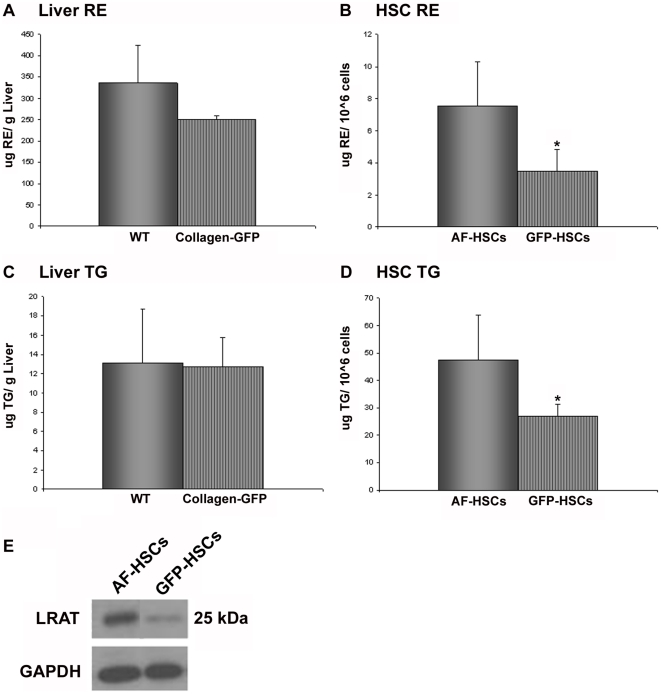
Total retinyl ester and triglyceride levels in liver and HSCs. (A) Retinyl ester (RE) levels in livers from WT and collagen-GFP mice, expressed as µg RE per gram liver weight. (B) Retinyl ester levels in AF-HSCs and GFP-HSCs, expressed as µg RE per million cells. (C) Triglyceride (TG) levels in livers from WT and collagen-GFP mice, expressed as µg TG per gram liver weight. Mice were fasted for 4 hours prior to sacrifice and tissue collection. (D) TG levels in AF-HSCs and GFP-HSCs, expressed as µg TG per million cells. Significance was determined by a Student's *t*-test, * p-value<0.05. (E) Protein levels of LRAT in AF-HSCs and GFP-HSCs. Experiment was repeated with similar results.

**Figure 4:**
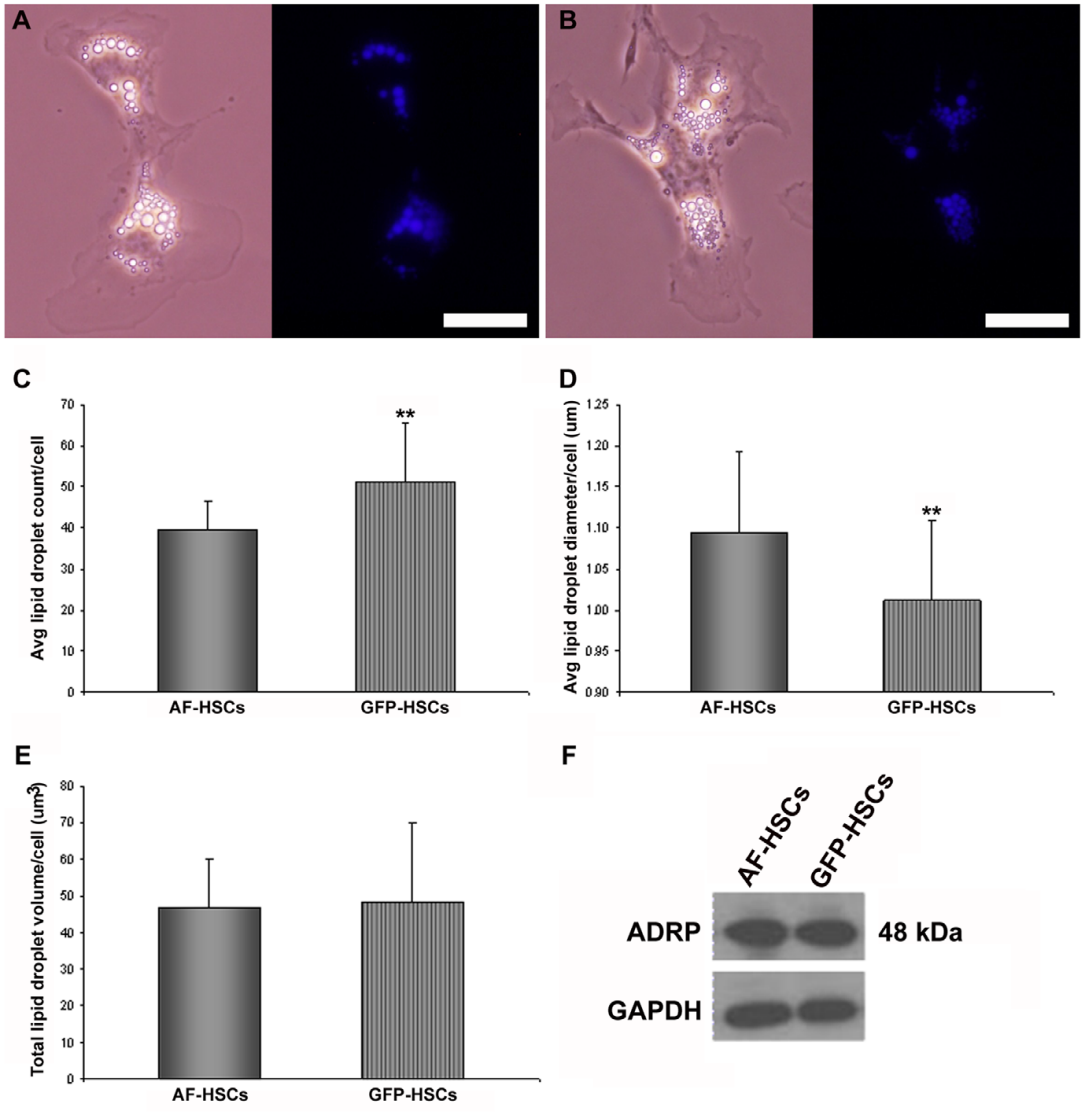
Assessment of lipid droplet content in AF-HSCs and GFP-HSCs after overnight culture. AF-HSCs and GFP-HSCs were isolated by FACS and incubated at 37°C overnight on glass bottom dishes. Phase contrast and fluorescent images were captured at 60× magnification for both AF-HSCs (A) and GFP-HSCs (B). Scale bars represent 30 µm. Average number of lipid droplets (C), average diameter (µm) of lipid droplets (D) and total lipid droplet volume (µm^3^) (E) per HSC are shown. (F) Protein levels of ADRP in AF-HSCs and GFP-HSCs. Experiment was repeated with similar results.

The elevated levels of markers of HSC activation prompted us to then examine whether the GFP-HSCs have less retinyl ester and triglyceride, which are the two predominant lipid species present in quiescent HSC lipid droplets [8]. We found that, while the livers of the WT and collagen-GFP mice have comparable levels of both lipid species (**Figures 3A and 3C**), the GFP-HSCs have significantly less retinyl ester (**Figure 3B**) and triglyceride (**Figure 3D**). To further assess lipid droplet content in these cells, AF- and GFP-HSCs were isolated by FACS and cultured overnight on glass plates. Lipid droplet quantity and size were assessed by phase contrast and fluorescent microscopy (**Figure 4A and 4B**). We found that GFP-HSCs have on average more lipid droplets per cell than AF-HSCs (**Figure 4C**); however, these droplets are smaller in size, determined by the average diameter of lipid droplets per cell measured in microns (**Figure 4D**). We also calculated the theoretical lipid droplet volume per cell, assuming a lipid droplet is a perfect sphere. Total lipid droplet volume per cell is the same in both populations (**Figure 4E**), and furthermore, the lipid droplet-associated protein adipose differentiation related protein (ADRP) is also expressed at equivalent levels in both AF- and GFP-HSCs (**Figure 4F**).

### Changes in retinoid- and lipid-related gene expression

**Figure 5:**
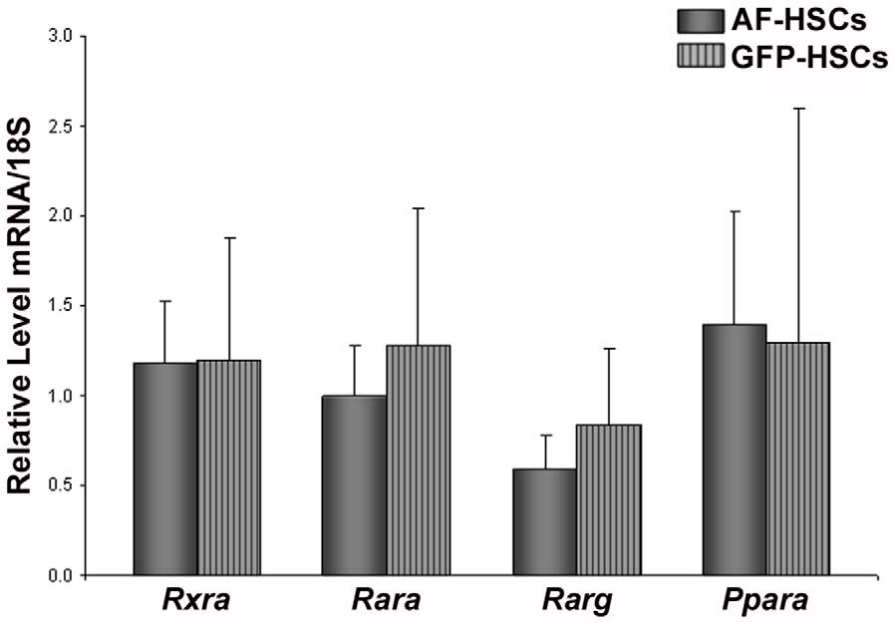
mRNA levels of retinoid- and lipid-related nuclear receptors. Relative mRNA expression levels of *Rxra*, *Rara*, *Rarg*, and *Ppara* in AF-HSCs (solid bars) and GFP-HSCs (stripped bars) are shown. Values are normalized to 18S and given as the mean ±1 S.D. for 5 AF and 5 GFP HSC isolations.

**Figure 6:**
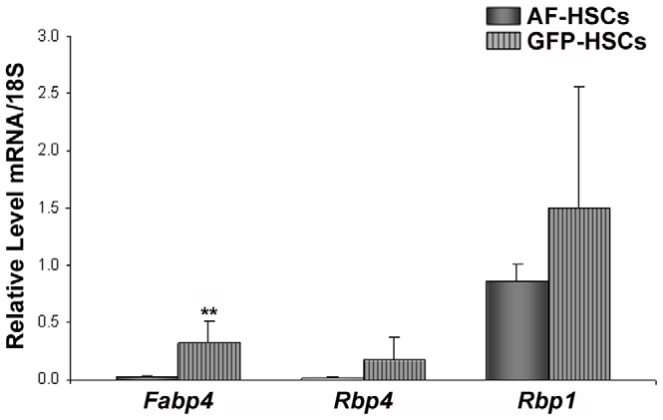
mRNA levels of retinol and fatty acid binding proteins. Relative mRNA expression levels of *Fabp4*, *Rbp4*, and *Rbp1* in AF-HSCs (solid bars) and GFP-HSCs (stripped bars) are shown. Values are normalized to 18S and given as the mean ±1 S.D. for 5 AF and 5 GFP HSC isolations.

**Figure 7:**
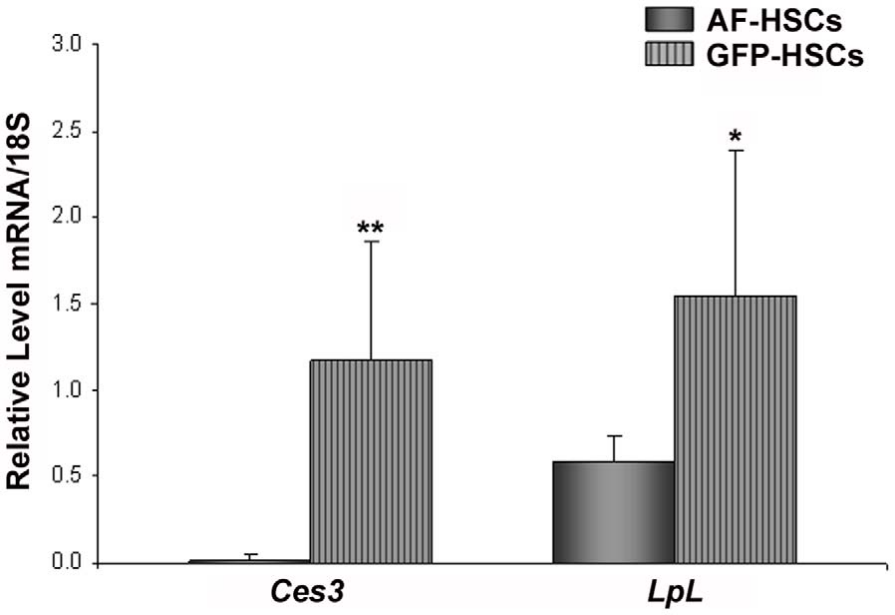
mRNA levels of lipid hydrolases. Relative mRNA expression levels of *Ces3* and *LpL* in AF-HSCs (solid bars) and GFP-HSCs (stripped bars) are shown. Values are normalized to 18S and given as the mean ±1 S.D. for 5 AF and 5 GFP HSC isolations. Significance was determined by a Student's *t*-test, * p-value<0.05 and ** p-value<0.01.

To better understand the lower levels of retinyl ester and triglyceride in GFP-HSCs, we focused our array analysis on retinoid- and lipid-related gene expression. The retinoid and lipid nuclear receptors were of high interest, as this group of genes represents a major route of gene regulation with many downstream effectors. The array data indicated that levels of the retinoid X receptor alpha (*Rxra*), retinoic acid receptor alpha and gamma (*Rara* and *Rarg*) and the peroxisome proliferator activated receptor alpha (*Ppara*) are lower in GFP-HSCs (**Table 4**). However, qRT-PCR analysis did not confirm significant differences in the expression of these genes in this population of cells (**Figure 5**). Similarly, the array suggested differential expression in the retinol- and fatty acid binding proteins, specifically retinol binding protein (*Rbp4*), cellular retinol binding protein 1 (*Rbp1*) and fatty acid binding protein 4 (*Fabp4*) (**Table 5**). qRT-PCR analysis of mRNA levels confirmed that *Fabp4* has significantly higher expression in the GFP-HSCs (**Figure 6**). The array additionally suggested that there may be significant gene transcriptional changes relating to lipid metabolism underlying the altered lipid content in the GFP-HSCs. There is elevated expression of two triglyceride lipases, diacylglycerol lipase, beta (*Daglb*) (**Table 6**) and arylacetamide deacetylase (*Aadac*) (**Table 7**). A number of phospholipases exhibited higher expression in the GFP-HSCs, including various members of the A and C subfamilies (**Table 7**). Notably, the array showed that carboxylesterase 3 (*Ces3*) expression is 82-fold higher in the GFP-HSCs (**Table 7**), and this elevation was confirmed by qRT-PCR of *Ces3* mRNA levels (**Figure 7**). This is interesting in light of the fact that a number of carboxylesterases have been proposed to be hepatic retinyl ester hydrolases [25], [26]. Also, we found via qRT-PCR analysis that lipoprotein lipase (*LpL*) mRNA expression is elevated in the GFP-HSCs (**Figure 7**).

**Table 4:** Retinoid- and Lipid-related Nuclear Receptors.

AFFY ID	Gene Name	Gene ID	FC	p-value
1454773_at	Retinoid X receptor alpha	*Rxra*	**0.5**	0.005977
1450180_a_at	Retinoic acid receptor, alpha	*Rara*	**0.4**	0.002350
1419416_a_at	Retinoic acid receptor, gamma	*Rarg*	**0.4**	0.003971
1425762_a_at	Retinoid X receptor alpha	*Rxra*	**0.4**	0.000323
1439675_at	Peroxisome proliferator activated receptor alpha	*Ppara*	**0.3**	0.004452

Differences in gene expression are reported as fold-change (FC) relative to AF-HSCs.

**Table 5:** Retinol and Fatty Acid Binding Proteins.

AFFY ID	Gene Name	Gene ID	FC	p-value
1451263_a_at	Fatty acid binding protein 4	*Fabp4*	**10.9**	0.001481
1417023_a_at	Fatty acid binding protein 4	*Fabp4*	**4.9**	0.003252
1416022_at	Fatty acid binding protein 5	*Fabp5*	**3.4**	0.002208
1426225_at	Retinol binding protein 4	*Rbp4*	**3.0**	0.022103
1416021_a_at	Fatty acid binding protein 5	*Fabp5*	**2.8**	0.000799
1450779_at	Fatty acid binding protein 7	*Fabp7*	**2.1**	0.014394
1425105_at	Retinol binding protein 3, interstitial	*Rbp3*	**0.5**	0.024528
1448754_at	Retinol binding protein 1, cellular	*Rbp1*	**0.5**	0.010727

Differences in gene expression are reported as fold-change (FC) relative to AF-HSCs.

**Table 6:** Lipases.

AFFY ID	Gene Name	Gene ID	FC	p-value
1430550_at	Lipase, family member M	*Lipm*	**6.1**	0.047718
1452398_at	Phospholipase C, epsilon 1	*Plce1*	**3.9**	0.000029
1417785_at	Phospholipase A1 member A	*Pla1a*	**3.5**	0.001223
1430700_a_at	Phospholipase A2, group VII (platelet-activating factor acetylhydrolase, plasma)	*Pla2g7*	**3.5**	0.002280
1457157_at	Phospholipase C-eta1a	*Plch1*	**2.6**	0.007681
1425338_at	Phospholipase C beta 4	*Plcb4*	**2.5**	0.007491
1436821_at	Phosphatidylinositol-specific phospholipase C, X domain containing 3	*Plcxd3*	**2.4**	0.031885
1437872_at	NAPE-PLD mRNA for N-acyl-phosphatidylethanolamine-hydrolyzing phospholipase D	*Napepld*	**2.3**	0.017539
1451970_at	Diacylglycerol lipase, beta	*Daglb*	**2.3**	0.011264
1435771_at	Phospholipase C beta 4	*Plcb4*	**2.1**	0.002450
1448558_a_at	Phospholipase A2, group IVA	*Pla2g4a*	**2.0**	0.001474
1424259_at	Lipase maturation factor 1	*Lmf1*	**0.5**	0.042273
1454619_at	Lipase maturation factor 2	*Lmf2*	**0.5**	0.000238
1416013_at	Phospholipase D family, member 3	*Pld3*	**0.4**	0.000290
1421261_at	Lipase, endothelial	*Lipg*	**0.4**	0.008316
1432928_at	Phospholipase C, delta 4	*Plcd4*	**0.4**	0.039780
1450188_s_at	Lipase, endothelial	*Lipg*	**0.4**	0.002089
1455448_at	Diacylglycerol lipase, alpha	*Dagla*	**0.4**	0.000027
1417433_at	Lysophospholipase 2	*Lypla2*	**0.2**	0.003292
1433949_x_at	Soluble PLA2-Ib precursor	*Pla2g1b*	**0.2**	0.018829
1450128_at	Phospholipase A2, group IIA	*-*	**0.1**	0.038422

Differences in gene expression are reported as fold-change (FC) relative to AF-HSCs.

**Table 7:** Esterases.

AFFY ID	Gene Name	Gene ID	FC	p-value
1449081_at	Carboxylesterase 3	*Ces3*	**82.2**	0.000234
1421218_at	Butyrylcholinesterase	*Bche*	**45.9**	0.001056
1436090_at	Ectonucleotide pyrophosphatase/phosphodiesterase 6	*Enpp6*	**13.7**	0.014343
1448813_at	Arylacetamide deacetylase (esterase)	*Aadac*	**10.9**	0.003532
1417300_at	Sphingomyelin phosphodiesterase, acid-like 3B	*Smpdl3b*	**5.3**	0.000398
1438665_at	Sphingomyelin phosphodiesterase 3, neutral	*Smpd3*	**5.1**	0.002101
1438785_at	Ectonucleotide pyrophosphatase/phosphodiesterase 6	*Enpp6*	**4.8**	0.004224
1452202_at	Phosphodiesterase 2A, cGMP-stimulated	*Pde2a*	**4.5**	0.006551
1422779_at	Sphingomyelin phosphodiesterase 3, neutral	*Smpd3*	**4.3**	0.041569
1447707_s_at	Phosphodiesterase 2A, cGMP-stimulated	*-*	**3.6**	0.026599
1416913_at	Esterase 1	*Es1*	**3.4**	0.044153
1417626_at	Phosphodiesterase 4D interacting protein	*-*	**2.8**	0.001982
1448136_at	Ectonucleotide pyrophosphatase/phosphodiesterase 2	*Enpp2*	**2.8**	0.004744
1424150_at	Glycerophosphodiester phosphodiesterase domain containing 5	*Gdpd5*	**2.6**	0.030611
1451857_a_at	Notum pectinacetylesterase homolog	*Notum*	**2.5**	0.015403
1417667_a_at	Phosphotriesterase related	*Pter*	**2.4**	0.000644
1427302_at	Ectonucleotide pyrophosphatase/phosphodiesterase 3	*Enpp3*	**2.3**	0.000140
1415894_at	Ectonucleotide pyrophosphatase/phosphodiesterase 2	*Enpp2*	**2.2**	0.001248
1437341_x_at	2′,3′-cyclic nucleotide 3′ phosphodiesterase	*-*	**0.5**	0.000609
1431913_a_at	Phosphodiesterase 3A, cGMP inhibited	*-*	**0.5**	0.000050
1435096_at	Resistance to inhibitors of cholinesterase 8 homolog B	*Ric8b*	**0.5**	0.001273
1422635_at	Acetylcholinesterase	*Ache*	**0.5**	0.004425
1437989_at	Phosphodiesterase 8B	*-*	**0.5**	0.000826
1421353_at	CAMP specific phosphodiesterase 7B	*Pde7b*	**0.4**	0.015231
1423810_at	Protein phosphatase methylesterase 1	*Ppme1*	**0.4**	0.000273
1418302_at	Palmitoyl-protein thioesterase 2	*Ppt2*	**0.4**	0.000061
1452897_at	Cell division cycle 2-like 5 (cholinesterase-related cell division controller)	*Cdc2l5*	**0.4**	0.000532
1421535_a_at	Phosphodiesterase 4A, cAMP specific	*Pde4a*	**0.4**	0.002163
1432490_a_at	Phosphodiesterase 10A	*-*	**0.4**	0.046869
1450453_a_at	CGMP-phosphodiesterase 6 gamma subunit	*Pde6g*	**0.4**	0.035910
1442700_at	Phosphodiesterase 4B, cAMP specific	*Pde4b*	**0.4**	0.000132
1451615_at	Carboxylesterase 8	*Ces8*	**0.3**	0.012629

Differences in gene expression are reported as fold-change (FC) relative to AF-HSCs.

### Cytochrome-mediated retinoid catabolism

**Figure 8:**
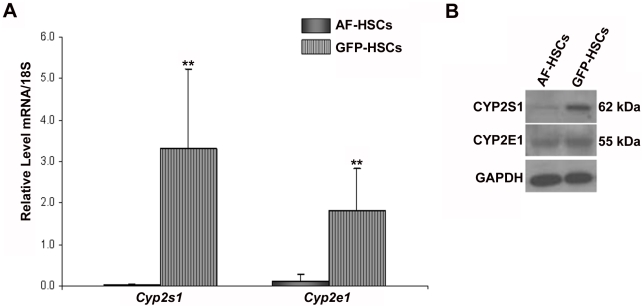
mRNA and protein expression of retinoid-metabolizing cytochrome P450 enzymes. (A) Relative mRNA expression levels of *Cyp2s1* and *Cyp2e1* in AF-HSCs (solid bars) and GFP-HSCs (stripped bars) are shown. Values are normalized to 18S and given as the mean ±1 S.D. for 5 AF and 5 GFP HSC isolations. Significance was determined by a Student's *t*-test, ** p-value<0.01. (B) Protein levels of CYP2S1 and CYP2E1 in AF-HSCs and GFP-HSCs. Experiments were repeated with similar results.

Since it is well established that HSC retinoids are lost upon cell activation, we hypothesized that the lower levels of retinyl ester in GFP-HSCs is a consequence of either increased mobilization of retinol from the HSCs or increased retinoid catabolism within the HSCs. Our data suggest that the first hypothesis is unlikely since we observe no differential expression of the retinoid-binding proteins in the GFP-HSCs (**Figure 6**), which are responsible for inter- and intracellular transport of retinoid in the body. To investigate the second hypothesis, we considered changes in gene expression in cytochrome P450 enzymes. The cytochromes (CYPs) are known to metabolize both exogenous compounds, including chemicals in the environment, carcinogens and drugs, and also endogenous compounds, including retinoids [27]. The array suggests that a number of CYPs are differentially expressed in GFP-HSCs (**Table 8**). However, we are specifically interested in CYPs that are known to metabolize retinoids and/or are proposed to play a role in HSC activation. Of the twenty five CYPs shown to be differentially expressed on the array, two meet these criteria: *Cyp2s1*, which has 32-fold higher expression in GFP-HSCs, and *Cyp2e1*, which has approximately 3-fold higher expression in GFP-HSCs (**Table 8**). We confirmed higher levels of *Cyp2s1* and *Cyp2e1* mRNA (**Figure 8A**) and in CYP2S1 protein (**Figure 8B**) in GFP-HSCs compared to AF-HSCs. CYP2E1 protein was unchanged (**Figure 8B**).

**Table 8:** Cytochrome P450 enzymes.

AFFY ID	Gene Name	Gene ID	FC	p-value
1416612_at	Cytochrome P450, family 1, subfamily b, polypeptide 1	*Cyp1b1*	**100.9**	0.000175
1428283_at	Cytochrome P450, family 2, subfamily s, polypeptide 1	*Cyp2s1*	**32.0**	0.004925
1460604_at	Cytochrome b reductase 1	*Cybrd1*	**23.6**	0.000016
1421075_s_at	Cytochrome P450, family 7, subfamily b, polypeptide 1	*Cyp7b1*	**17.1**	0.000044
1417507_at	Cytochrome b-561	*Cyb561*	**13.2**	0.000421
1421074_at	Cytochrome P450, family 7, subfamily b, polypeptide 1	*Cyp7b1*	**10.2**	0.000009
1425040_at	Cytochrome b reductase 1	*Cybrd1*	**9.2**	0.000095
1416613_at	Cytochrome P450, family 1, subfamily b, polypeptide 1	*Cyp1b1*	**8.9**	0.000029
1419559_at	Cytochrome P450, family 4, subfamily f, polypeptide 14	*Cyp4f14*	**8.4**	0.000002
1422533_at	Cytochrome P450, family 51	*Cyp51*	**4.7**	0.000374
1436778_at	Cytochrome b-245, beta polypeptide	*Cybb*	**4.4**	0.000743
1422978_at	Cytochrome b-245, beta polypeptide	*Cybb*	**4.0**	0.001173
1444138_at	Cytochrome P450, family 2, subfamily r, polypeptide 1	*Cyp2r1*	**3.9**	0.016331
1436779_at	Cytochrome b-245, beta polypeptide	*Cybb*	**3.8**	0.003131
1450646_at	Cytochrome P450, family 51	*Cyp51*	**3.4**	0.001651
1415994_at	Cytochrome P450, family 2, subfamily e, polypeptide 1	*Cyp2e1*	**2.9**	0.006021
1422534_at	Cytochrome P450, family 51	*Cyp51*	**2.8**	0.010709
1418780_at	Cytochrome P450, family 39, subfamily a, polypeptide 1	*Cyp39a1*	**2.2**	0.038740
1418709_at	Cytochrome c oxidase, subunit VIIa 1	*Cox7a1*	**2.1**	0.017825
1440211_at	Cytochrome P450, family 2, subfamily j, polypeptide 11	*Cyp2j11*	**0.5**	0.026833
1416933_at	P450 (cytochrome) oxidoreductase	*Por*	**0.5**	0.002868
1430452_at	Cytochrome P450, family 20, subfamily A, polypeptide 1	*Cyp20a1*	**0.5**	0.000408
1416112_at	Cytochrome c oxidase, subunit VIIIa	*Cox8a*	**0.4**	0.000037
1450752_at	Cytochrome c, testis	*Cyct*	**0.3**	0.011154
1425645_s_at	Cyp2b10-like pseudogene	*Cyp2b10*	**0.3**	0.040338

Differences in gene expression are reported as fold-change (FC) relative to AF-HSCs.

**Figure 9:**
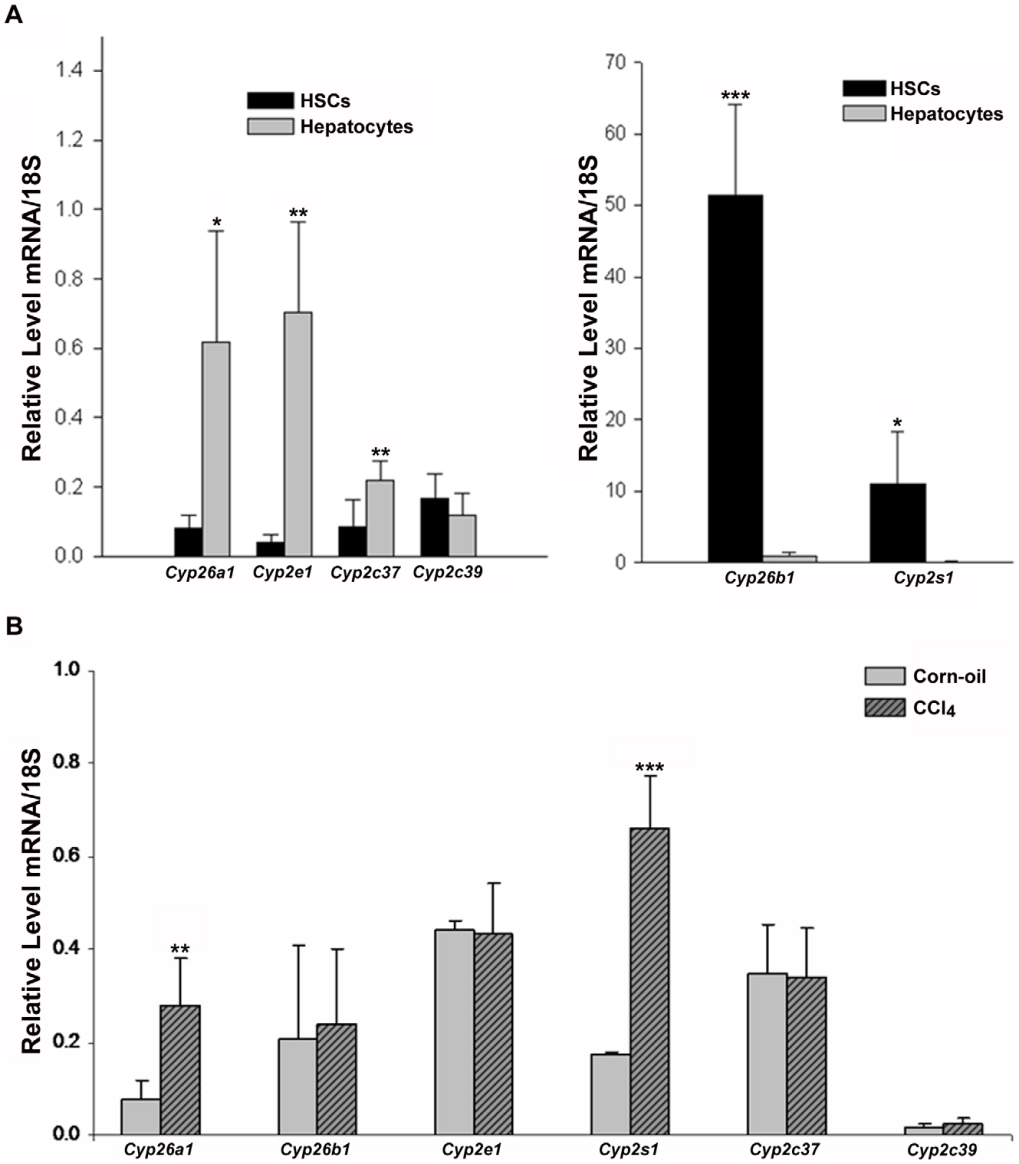
*Cyp2s1* is a retinoid-catabolizing CYP both highly expressed in HSCs and induced in hepatic fibrosis. (A) HSCs and hepatocytes were isolated from male WT C57 mice at 3 months age. Relative mRNA expression levels of *Cyp26a1*, *Cyp2e1*, *Cyp2c37*, and *Cyp2c39* (left panel) and *Cyp26b1* and *Cyp2s1* (right panel) are shown. (B) WT C57 male mice were given weekly injections of either corn oil or 0.5 ul CCl_4_/g B.W. administered in corn oil for 4 weeks and then sacrificed. Relative *Cyp* mRNA levels in whole liver homogenates were measured. Values are normalized to 18S and given as the mean ±1 S.D. Significance was determined by a Student's *t*-test, * p-value<0.05, ** p-value<0.01, *** p-value<0.001.

CYP2S1 is a recently-identified cytochrome enzyme that has been shown to be expressed highly in the lung, small intestine and spleen and to be inducible by dioxin, which acts via the aryl hydrocarbon receptor [28], [29]. It has also been reported to both metabolize and be induced by all-*trans*-retinoic acid [30]. The literature does not definitively establish the expression of CYP2S1 in the liver; CYP2S1 expression has been detected in the liver, albeit at very low levels [31], [32]. More recently, CYP2S1 was shown to be expressed in human HSCs [33]. To further elucidate the expression of CYP2S1 in the mouse liver, we isolated both hepatocytes and HSCs and measured mRNA levels of 6 CYPs, which were chosen based on their known liver expression, roles in retinoid metabolism and/or proposed roles in hepatic disease. We found that while *Cyp2e1* is more highly expressed in hepatocytes, *Cyp2s1* is highly enriched in HSCs (**Figure 9A**).

Having shown *Cyp2s1* to be highly expressed in HSCs and elevated in collagen-expressing HSCs relative to the non-collagen-expressing population, we became interested in whether this cytochrome might play a role in the early events of hepatic fibrosis, when HSCs activate and lose their lipid droplet content. To address this question, we injected WT C57 mice around 3 months of age with either CCl_4_ (0.5 µl/g body weight) or corn oil vehicle control once a week for 4 weeks and assessed changes in mRNA expression of the 6 CYPs discussed above. Total hepatic retinol (retinol+retinyl ester) levels are significantly decreased in mice receiving CCl_4_ injections (**Figure S2**), confirming successful HSC activation in these mice. We found that hepatic levels of *Cyp26a1* and *Cyp2s1* were both elevated when mice were treated with CCl_4_, by approximately 4-fold in each case (**Figure 9B**). Thus, we identify *Cyp2s1* as the only retinoid-catabolizing CYP that is both highly expressed in HSCs and induced in CCl_4_-mediated HSC activation. We propose that CYP2S1 plays a role in the catabolism of HSC retinoid content, contributing to the lower retinyl ester and lipid droplet content in both collagen-expressing HSCs and HSCs activated *in vivo* by CCl_4_ exposure.

## Discussion

The idea that there is heterogeneity and plasticity in the HSC population in the liver is not new, but has not previously been well-studied. Studies in the early 1990s introduced this idea, utilizing the HSC marker desmin to show that there is a population of desmin-negative, lipocyte-like cells in the liver [34] and that these HSCs are vitamin A-deficient [35]. Our study extends these earlier reports and the proposition that not all HSCs in the liver are equivalent by using different methodologies to isolate two distinct populations of HSCs from the liver and to characterize their morphological and biochemical properties. The different methods of cell isolation we used take advantage of distinct properties of these cells: the first method relies on HSC lipid droplet vitamin A content and the second on HSC collagen I gene expression.

The standard method of HSC isolation employs a Nycodenz density gradient to separate non-parenchymal cells from a liver digest where hepatocytes have been destroyed by exposure to pronase [9], [21], [22]. The low buoyant density of the numerous lipid droplets present in the HSCs (see **Figure 4**) allows these cells to float to the top of the gradient and thereby be separated from the other less lipid-rich cell types present in the liver. To obtain a vitamin A-enriched population of HSCs, we additionally conducted FACS on the nycodenz-purified cells. Vitamin A autofluoresces upon excitation at 350 nm, so exposure to this wavelength of light allowed us to collect a population of cells that is enriched in vitamin A. We also employed a second FACS-based method to isolate HSCs. This involved the use of collagen-GFP mice in which GFP expression is driven by the *Col1a1* promoter [16]. Our data confirm that collagen I expression in the liver is confined to the HSCs, with GFP expression co-localizing exclusively with desmin-positive cells. Our data additionally show that the GFP-based method of HSC isolation yields a population of cells with equivalent desmin expression to the conventional Nycodenz-based method. Thus, we demonstrate for the first time a novel method of HSC isolation that yields cells of identical purity to cells isolated by the conventional Nycodenz-based method.

When a liver is confronted with acute injury, a wound-healing response is initiated which ultimately returns the liver to its healthy state; however, when the liver is confronted with chronic injury, the result is often hepatic fibrosis, which is the production of excess fibrous connective tissue as a reparative process to contain the site of injury [13], [14]. In the early stages of hepatic fibrosis, the HSCs undergo a process of activation, whereby they transition from a quiescent, lipocyte-like state to a myofibrosblastic, contractile phenotype. Activated HSCs express high levels of COL1a1 and ACTA2, which are key components of the extracellular matrix. Other key proliferative, fibrogenic and contractile stimuli in hepatic fibrosis are PDGF-C, TGF-β and EDN1, respectively [36]. Induction of ACTA2 is the single most reliable marker of HSC activation because it is absent from other resident liver cells in either normal or injured liver; furthermore, its expression connotes a contractile phenotype [37]. Our data show that GFP-HSCs have significantly elevated levels of both ACTA2 mRNA and protein, indicating that this is a population of HSCs in early activation. Because the livers from which these cells were isolated were not experimentally injured or chemically induced, these findings suggest that there is a population of pre-activated HSCs in the liver under physiological conditions. This idea supports earlier studies in transgenic mice, in which GFP was driven by the collagen I promoter and red fluorescent protein (RFP) was driven by the *Acta2* promoter [38]. It was shown that there are mixed populations of activated HSCs, such that some populations expressed GFP or RFP alone and some expressed both. There may be two explanations for this phenomenon. One possibility is that these cells are partially activated in response to a local hepatic insult, which could involve the natural microbiome (perhaps, the gut flora) of the organism and elicitation of an LPS response; alternatively, the HSC population in a healthy liver may be in constant flux, such that there is a cyclical turn-over of quiescent and activated HSCs. We believe that the second possibility is more likely and that our finding that there exists a pre-activated population of HSCs in a healthy liver reflects the dynamic physiology of these cells, which ensures that there will always be a subset of the population ready to respond to hepatic injury.

Lipid droplets are indispensable, metabolically active organelles found ubiquitously across species (including plants, animals and yeasts) and also within different animal cell types (adipocytes, macrophages, hepatocytes, hepatic stellate cells, etc.) [39]. Thus, lipid droplets are versatile and diverse. Lipid droplets found in different cell types have different lipid and protein compositions. Cell-specific localization combined with unique composition dictates regulation of lipid droplet formation and maintenance in a cell type-specific manner. There is a strong correlation between the predominant lipid species in the droplet and the enzyme(s) responsible for synthesis of the lipid. For instance, an adipocyte lipid droplet, which is composed primarily of triglyceride synthesized by DGAT1 (diacylglycerol acyltransferase 1) and/or DGAT2, will not be formed when these two enzymes are not expressed [40]. Similarly, the HSC lipid droplet has a unique retinoid and lipid content, not found in other cell types in the body [8], [9]. The retinyl ester content is dependent on expression of LRAT, which is the only hepatic enzyme capable of retinyl ester synthesis *in vivo* [10]. We show that when LRAT expression is diminished, these lipid droplets are smaller in size, suggesting degradation. Importantly, it is also established that as HSCs undergo activation, they lose their retinyl ester-containing lipid droplets; though it is not known if this loss is a cause or consequence of the activation process. Thus, our findings correlate strongly with the events known to occur in the early stages of HSC activation. LRAT expression is low, retinyl ester levels are decreased, and there are marked alterations in the HSC lipid droplets.

Our observations regarding the different lipid droplet content in the GFP-HSCs additionally allows us to hypothesize about the events taking place in the very early stages of HSC activation. It is important to emphasize that the GFP-HSCs have not been experimentally induced, so we distinguish these cells as “pre-activated” HSCs: they are not fully activated, but they display characteristic features of such cells, including decreased retinyl ester levels and elevated expression of ACTA2 mRNA and protein. A priori, we would expect the early events of HSC activation to follow one of the following three scenarios: (i) lipid droplets progressively decrease in number, eventually leading to the complete loss of droplets; (ii) lipid droplets progressively decrease in size, eventually leading to the complete loss of droplets; or (iii) lipid droplets are initially broken-down into many more, smaller droplets – increase in number, decrease in size - and then they progressively decrease in number until none remain in the cell. Interestingly, our data provide strong evidence for the third, more complex scenario. We show that the GFP-HSCs, a population of uninduced but collagen-expressing cells, have more lipid droplets than the AF-HSCs, and these droplets are smaller in size, as determined by a decrease in average diameter. Our data is in good agreement with other studies investigating lipid droplet dissolution in other cellular systems. It is known that in the first several hours of hormone-stimulated lipolysis in 3T3-L1 adipocytes, the lipid droplets present in these cells are fragmented and dispersed into microlipid droplets, which significantly increases the surface area available for lipase activity [41]–[43]. A similar pattern of lipid droplet dissolution was reported for rat adipocytes, in which lipolysis was stimulated by electroporation of a constitutively active β-1 adrenergic receptor into the fat pads of the animals [44]. It will be necessary for future studies to confirm this sequence of events in HSCs directly with real-time tracking of lipid droplet remodeling in response to HSC activation. It will also be necessary to define the contribution of the lipid droplet-associated proteins, such as ADRP, to these processes. Our data shows ADRP protein expression is not different in the GFP-HSCs, but its distribution in the cell and on the lipid droplets might be changed as a consequence of the beginning stages of lipolysis.

Our data also establish decreased triglyceride levels, along with the differential expression of many lipid metabolism-related genes, in GFP-HSCs. This is not surprising since triglyceride is the second most predominant lipid species in HSC lipid droplets (31.7%), only slightly less than retinyl ester (39.5%) [8], [9]. It is likely that when retinyl esters are lost as a result of activation, the HSC lipid droplet is targeted and signaled to breakdown, and thus, synthesis of the non-retinoid lipids is down-regulated. This is analogous to the idea discussed above that when the predominant lipid found in a particular droplet is not synthesized, the droplet will not form. In the case of the GFP-HSCs we are studying, some retinyl ester is still being made since LRAT is still expressed; thus, the droplets are present, but they are smaller in size. The decrease in HSC triglyceride levels could be a consequence of either decreased triglyceride synthesis or increased triglyceride catabolism. The array data suggest that triglyceride synthesis is not markedly different in the GFP-HSCs. There are no genes involved in triglyceride biosynthesis with significant differential expression in the GFP-HSCs. DGAT1 and DGAT2 are known triglyceride synthesizing enzymes in the liver [45], [46], but expression levels of both are the same in the two HSC populations (data not shown). There are, however, many differences in expression in genes involved with triglyceride breakdown, classified as either lipases or esterases. We observe increased expression of two triglyceride lipases and a number of phopholipases. Phospholipids comprise approximately 6% of lipids in HSC lipid droplets [8], [9]; thus, the increase in phopsholipid catabolism supports the idea that when retinyl ester is low, the non-retinoid lipids are degraded, as well. We also observe a large increase in expression of *Ces3*. This is interesting in light of the fact that other carboxylesterases have recently been proposed as potential retinyl ester hydrolases in HSCs, including ES-4 and -10 [47], [48]. Our findings suggest that Ces3 might be a retinyl ester hydrolase in activated HSCs, but more work will need to be done to confirm its HSC expression and role in retinyl ester hydrolysis. Similarly, we find elevated levels of *LpL* in the GFP-HSC population. Mello et al. have shown that LpL is expressed at very low levels in hepatocytes and HSCs, but its expression is induced 32-fold in activated HSCs [47]. Thus, the elevated levels of *LpL* provide further evidence that the GFP population are pre-activated HSCs. Overall, the highly altered lipid metabolism in the GFP-HSCs is very interesting since we still do not know much about the specific lipid species that populate HSC lipid droplets, particularly with regard to the exact fatty acyl compositions of the lipids in these cells. All we currently have is a fairly crude understanding of the breakdown of retinyl ester, triglyceride, cholesterol, phospholipids, and free fatty acids [8], [9]. Thus, future work in this area will prove to be very exciting with the potential of using the newly-emerging field of lipidomics and the use of LC/MS/MS for these characterizations.

The cytochrome P450's are a superfamily of enzymes known to metabolize both exogenous compounds, including chemicals in the environment, carcinogens and drugs, and also endogenous compounds, including retinoids [27]. We were interested in studying six cytochromes in particular, based on either their expression in the liver and/or proposed roles in different types of liver disease. The CYP26s (including CYP26A1, B1 and C1) are thought to be the major retinoid-catabolizing CYPs in the body [49]–[52]. They are known to metabolize all-*trans*-retinoic acid and additionally, to be retinoic acid-inducible. CYP26A1 and B1 are expressed in the mouse liver, but C1 is not. CYP2E1 is notable for its role in alcoholic liver disease and for its release of reactive oxygen species, which are known to promote fibrogenesis in HSCs [53], [54]. CYP2S1 is a relatively newly-identified CYP and thus is not well-studied; however, it has been reported to metabolize retinoic acid [28]–[30]. The last two, CYPs 2C37 and 2C39, have liver-specific expression [55]. Additionally, 2C39 has been reported to metabolize retinoic acid [56]. Of these six CYPs, only CYP2S1 was shown on the array, by qRT-PCR and by western blot to be elevated in the GFP-HSCs. Previous reports in the literature suggest that CYP2S1 has very low expression in the liver [31], [32]. Our data show that *Cyp2s1* is highly enriched in the HSCs, which comprise only 6–8% of cells in the liver and contain 1% of hepatic protein [6], [37]. Thus, expression in whole liver homgenates will be masked by the presence of hepatocytes, Kupffer cells and endothelial cells. Furthermore, we show that *Cyp2s1* expression is elevated *in vivo* by carbon tetrachloride-mediated induction of hepatic fibrosis. Thus, we propose that CYP2S1-mediated retinoid catabolism may play a role in the loss of lipid droplets and retinyl ester content in HSCs undergoing activation in the early stages of hepatic fibrosis.

In conclusion, using FACS-based methods of HSC isolation, we show that there are distinct populations of HSCs in a normal mouse liver. The GFP-isolated HSCs have altered lipid droplet content and display distinct biochemical properties, determined by differential gene and protein expression profiles and differential capacities for retinyl ester and triglyceride storage in their lipid droplets. Specifically, we show that the GFP-HSCs have: (i) increased expression of typical markers of HSC activation; (ii) significantly decreased retinyl esters, accompanied by reduced LRAT protein expression; (iii) significantly less triglycerides; (iv) increased expression of genes associated with lipid catabolism, i.e. hydrolases and esterases; and (v) an increase in expression of the retinoid-catabolizing CYP, CYP2S1. Our data suggest that there is heterogeneity in the HSC population in normal, uninjured liver. A subset of the HSC population may serve as “primers” for activation, such that they are the first responders in an injured liver.

## Supporting Information

Figure S1 **Quality assessment of samples used in microarray analysis.** Gene microarray analysis was conducted on populations of AF-HSCs isolated from 6 mice and GFP-HSCs isolated from 5 mice. cDNA purified from these populations was run on Affymetrix Mouse Genome 430 2.0 Arrays, and data was analyzed using GeneSifter software. (A) Levels of desmin mRNA normalized to 18S. A Student's *t*-test was used to analyze for statistically significant differences between groups. Groups were considered to be significantly different when p<0.05. (B) Boxplot summary showing the maximum and minimum values, 1^st^ and 3^rd^ quartiles, and medians of the AF and GFP groups. (C) Scatter plot summary showing the spread of the data points around the line of identity. All genes over-expressed in the GFP group are shown in red; genes over-expressed in the AF group are shown in green.
(TIF)

Figure S2 **Total retinol levels in the liver of mice treated with CCl_4_.** WT C57 male mice were given weekly injections of either corn oil or 0.5 ul CCl_4_/g B.W. administered in corn oil for 4 weeks and then sacrificed. Total retinol (retinol+retinyl ester) levels are shown, expressed as nmol total retinol per gram of liver. Significance was determined by a Student's *t*-test, * p-value<0.05.
(TIF)

Table S1 ABI primers used for qRT-PCR analysis. Commercially available primers purchased from Applied Biosystems (ABI) are shown with the ABI accession number for all genes qRT-PCR analysis was conducted on.
(DOC)
