# Supplementary figures and images for: Distinct Populations of Hepatic Stellate Cells in the Mouse Liver Have Different Capacities for Retinoid and Lipid Storage
Source: PLoS One. 2011 Sep 16;6(9):e24993. doi: 10.1371/journal.pone.0024993 (PMC3174979; doi:10.1371/journal.pone.0024993)

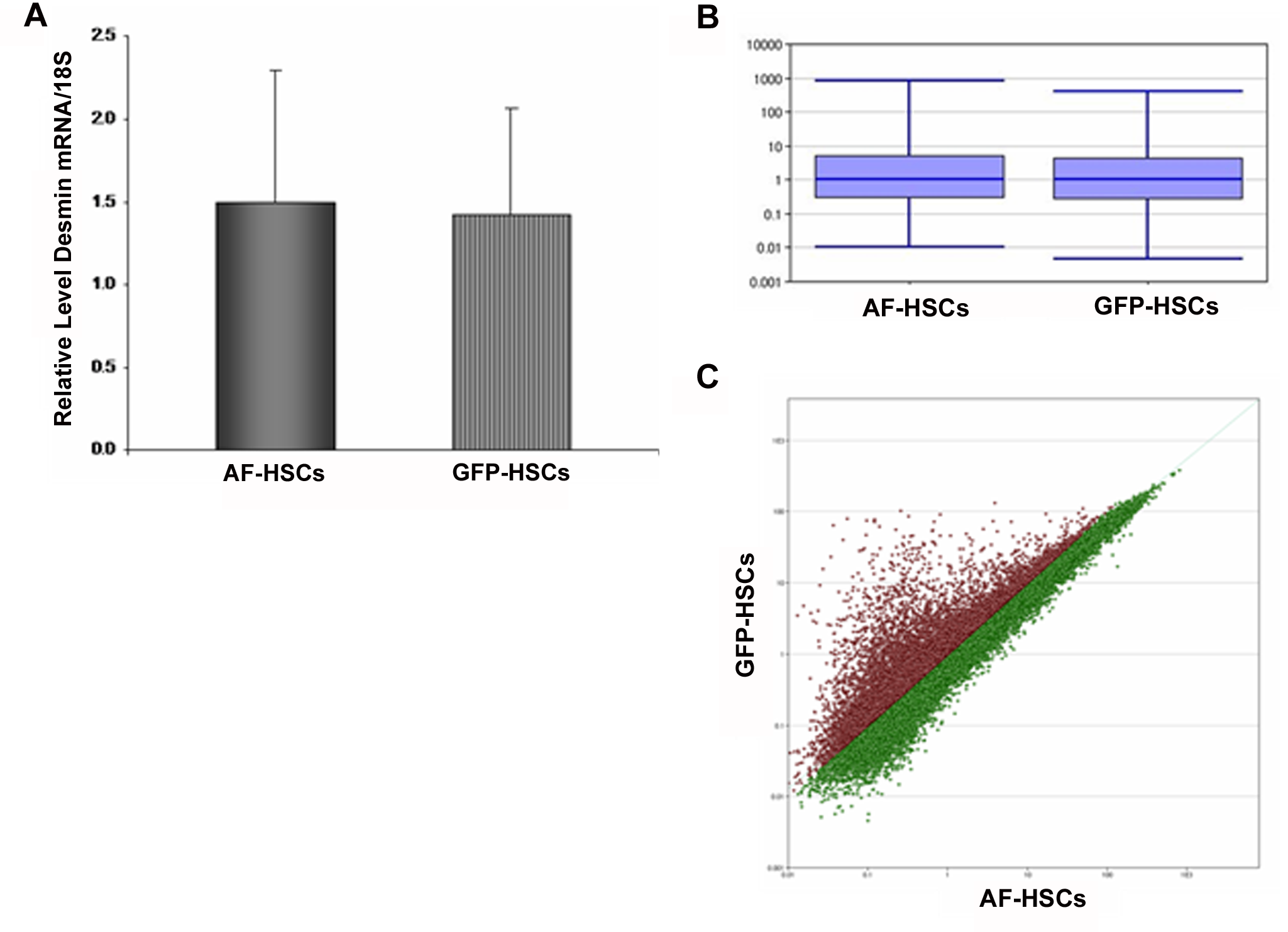

Supplement: Figure S1 — Quality assessment of samples used in microarray analysis. Gene microarray analysis was conducted on populations of AF-HSCs isolated from 6 mice and GFP-HSCs isolated from 5 mice. cDNA purified from these populations was run on Affymetrix Mouse Genome 430 2.0 Arrays, and data was analyzed using GeneSifter software. (A) Levels of desmin mRNA normalized to 18S. A Student's t-test was used to analyze for statistically significant differences between groups. Groups were considered to be significantly different when p<0.05. (B) Boxplot summary showing the maximum and minimum values, 1st and 3rd quartiles, and medians of the AF and GFP groups. (C) Scatter plot summary showing the spread of the data points around the line of identity. All genes over-expressed in the GFP group are shown in red; genes over-expressed in the AF group are shown in green. (TIF) [file pone.0024993.s001.tif]

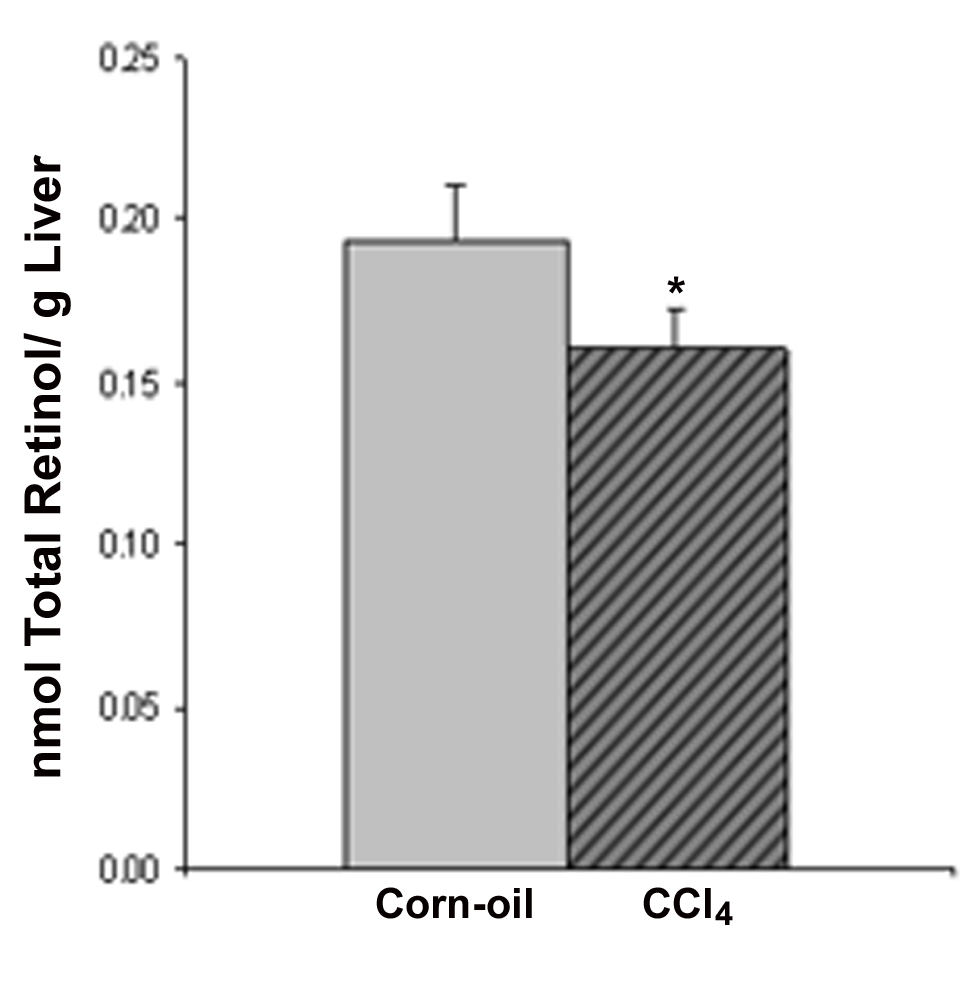

Supplement: Figure S2 — Total retinol levels in the liver of mice treated with CCl4. WT C57 male mice were given weekly injections of either corn oil or 0.5 ul CCl4/g B.W. administered in corn oil for 4 weeks and then sacrificed. Total retinol (retinol+retinyl ester) levels are shown, expressed as nmol total retinol per gram of liver. Significance was determined by a Student's t-test, * p-value<0.05. (TIF) [file pone.0024993.s002.tif]
