# Supplementary material for: Distinct Populations of Hepatic Stellate Cells in the Mouse Liver Have Different Capacities for Retinoid and Lipid Storage
Source: PLoS One. 2011 Sep 16;6(9):e24993. doi: 10.1371/journal.pone.0024993 (PMC3174979; doi:10.1371/journal.pone.0024993)
Supplement: Table S1 — ABI primers used for qRT-PCR analysis. Commercially available primers purchased from Applied Biosystems (ABI) are shown with the ABI accession number for all genes qRT-PCR analysis was conducted on. (DOC) [file pone.0024993.s003.doc]

**Table S1. ABI primers used for qRT-PCR analysis.**

| **ABI Primer** | **ABI Accession No.** |
| --- | --- |
| Desmin | Mm00802455_m1 |
| Col1a1 | Mm00801666_g1 |
| Acta2 | Mm00725412_s1 |
| Pdgfc | Mm00480205_m1 |
| Tgfb2 | Mm01178820_m1 |
| Edn1 | Mm00438656_m1 |
| Rxra | Mm01332431_m1 |
| Rara | Mm00436264_m1 |
| Rarg | Mm00441083_m1 |
| Ppara | Mm00440939_m1 |
| Fabp4 | Mm00445880_m1 |
| Rbp4 | Mm00803266_m1 |
| Rbp1 | Mm00441119_m1 |
| Ces3 | Mm00474816_m1 |
| LpL | Mm00434764_m1 |
| Cyp2s1 | Mm00512037_m1 |
| Cyp2e1 | Mm00491127_m1 |
| Cyp26a1 | Mm00514486_m1 |
| Cyp26b1 | Mm00558507_m1 |
| Cyp2c37 | Mm00833845_m1 |
| Cyp2c39 | Mm00656110_gH |

Commercially available primers purchased from Applied Biosystems (ABI) are shown with the ABI accession number for all genes qRT-PCR analysis was conducted on.
